# N6-Methyladenosine Methyltransferase METTL3 Promotes Angiogenesis and Atherosclerosis by Upregulating the JAK2/STAT3 Pathway via m6A Reader IGF2BP1

**DOI:** 10.3389/fcell.2021.731810

**Published:** 2021-12-07

**Authors:** Guo Dong, Jiangbo Yu, Gaojun Shan, Lide Su, Nannan Yu, Shusen Yang

**Affiliations:** ^1^ Department of Cardiovascular, The First Affiliated Hospital of Harbin Medical University, Harbin, China; ^2^ Department of Ophthalmology, The First Affiliated Hospital of Harbin Medical University, Harbin, China

**Keywords:** atherosclerosis, METTL3, JAK2, STAT3, IGF2BP1

## Abstract

Atherosclerosis (AS) is a life-threatening vascular disease. RNA N6-methyladenosine (m6A) modification level is dysregulated in multiple pathophysiologic processes including AS. In this text, the roles and molecular mechanisms of m6A writer METTL3 in AS progression were explored *in vitro* and *in vivo*. In the present study, cell proliferative, migratory, and tube formation capacities were assessed through CCK-8, Transwell migration, and tube formation assays, respectively. RNA m6A level was examined through a commercial kit. RNA and protein levels of genes were measured through RT-qPCR and western blot assays, respectively. VEGF secretion level was tested through ELISA assay. JAK2 mRNA stability was detected through actinomycin D assay. The relationship of METTL3, IGF2BP1, and JAK2 was investigated through bioinformatics analysis, MeRIP, RIP, RNA pull-down, and luciferase reporter assays. An AS mouse model was established to examine the effect of METTL3 knockdown on AS development *in vivo*. The angiogenetic activity was examined through chick chorioallantoic membrane assay *in vivo*. The results showed that METTL3 was highly expressed in ox-LDL-induced dysregulated HUVECs. METTL3 knockdown inhibited cell proliferation, migration, tube formation, and VEGF expression/secretion in ox-LDL-treated HUVECs, hampered AS process *in vivo*, and prevented *in vivo* angiogenesis of developing embryos. METTL3 positively regulated JAK2 expression and JAK2/STAT3 pathway in an m6A dependent manner in HUVECs. IGF2BP1 positively regulated JAK2 expression through directly binding to an m6A site within JAK2 mRNA in HUVECs. METTL3 knockdown weakened the interaction of JAK2 and IGF2BP1. METTL3 exerted its functions through JAK2/STAT3 pathway. In conclusion, METTL3 knockdown prevented AS progression by inhibiting JAK2/STAT3 pathway via IGF2BP1.

## Introduction

Atherosclerosis (AS), characterized by the deposition of lipids and other materials in and on the artery walls (AS plaques), is closely associated with the increased risks of multiple life-threatening cardiovascular diseases such as stroke, heart attack, and myocardial infarction (Khatana et al., 2020; Munjal and Khandia, 2020). The functional and structural integrity of endothelium is indispensable for in the maintenance of vascular homeostasis, and the dysfunction of vascular endothelial cells (all maladaptive alterations in the functional phenotypes) has been suggested to be a contributor to the initiation and progression of AS (Gimbrone and García-Cardeña, 2016; Brown et al., 2017). Oxidized low-density lipoprotein (ox-LDL) is a major risk factor for AS (Yang et al., 2012). Ox-LDL can regulate AS development through multiple mechanisms, including inducing the activation and dysfunction of endothelial cells (Pirillo et al., 2013; Di Pietro et al., 2016; Khatana et al., 2020). A deep insight into the molecular mechanisms underlying ox-LDL-induced endothelial dysfunction might contribute to a better understanding of the pathogenesis of AS.

N6-methyladenosine (m6A) is the most common modification in eukaryotic RNA transcripts (Wu et al., 2016). M6A modification can influence gene expression by regulating almost all aspects of RNA metabolism such as RNA stability, splicing, translation, and transport (Wu et al., 2016; He and He, 2021). Moreover, m6A-mediated gene regulation is implicated in various physiological and pathological processes (Lence et al., 2019; Jiang, et al., 2021). Additionally, it has been reported that the m6A methylation modification level is dysregulated in AS lesions and is associated with AS development (Jian et al., 2020; Quiles-Jiménez et al., 2020; Chen et al., 2021a). M6A modification is catalyzed by m6A methyltransferases (m6A writers), removed by m6A demethylases (m6A erasers), and recognized by m6A binding proteins (m6A readers) (Robinson et al., 2019; Jiang, et al., 2021). METTL3, an m6A writer, is involved in the regulation of multiple biological processes (e.g., proliferation, migration, and apoptosis) and disease (e.g., cancer, ischemic heart disease, and diabetes) progression (Liu et al., 2020a; Zheng et al., 2019). Moreover, METTL3 has been reported to be related to the dysfunction of vascular endothelium (Wang et al., 2020a; Yao et al., 2020). For instance, Yao et al*.* demonstrated that METTL3 expression was notably increased in hypoxia-exposed HUVECs compared to the normoxic group (Yao et al., 2020). METTL3 knockdown weakened the proliferative, migratory, and tube formation capacities of HUVECs *in vitro* and hampered retinal and corneal angiogenesis *in vivo* (Wang et al., 2020a; Yao et al., 2020). However, the roles and molecular basis of METTL3 in the development of AS are poorly defined.

In this text, the influence of METTL3 knockdown on ox-LDL-induced endothelial cell dysfunction, AS development, and angiogenesis along with related m6A-dependent regulatory mechanisms was investigated *in vitro* and *in vivo*.

## Materials and Methods

### Cell Culture

HUVECs were purchased from American Type Culture Collection (ATCC, Manassas, Virginia, United States) and maintained in endothelial cell medium (ScienCell Research Laboratories, San Diego, CA, United States) containing 5% fetal bovine serum (FBS; ScienCell Research Laboratories), 1% endothelial cell growth supplement (ECGS; ScienCell Research Laboratories) and 1% penicillin/streptomycin solution (ScienCell Research Laboratories). HUVECs from passages two to four were used in our project.

### Reagents and Cell Transfection

Ox-LDL was obtained from Biomedical Technologies, Inc. (Stoughton, MA, United States). Oil red O was obtained from Sigma-Aldrich, Inc. (St. Louis, MO, United States). Small interference RNAs (siRNA) targeting IGF2BP1 (si-IGF2BP1#1, si-IGF2BP1#2, and si-IGF2BP1#3), METTL3 (si-METTL3#1, si-METTL3#2, and si-METTL3#3), and a scrambled siRNA (si-NC) were obtained from GenePharma Co., Ltd. (Shanghai, China). The siRNA sequences were shown in Table 1. The recombinant plasmid expressing JAK2 (pcDNA-JAK2) or IGF2BP1 (pcDNA-IGF2BP1) was synthesized by GeneCreate Biological Engineering Co., Ltd. Plasmids or siRNAs were transfected into HUVECs using jetPRIME transfection reagent (Poluplus-transfection Inc., New York, NY, United States) according to the instructions of the manufacturer. For double transfection, DNA (2 μg) and siRNA (final concentration: 50 nM) were mixed and co-incubated with 5 μl jetPRIME reagent for 15 min at room temperature in 6-well plates. Next, the transfection mix was added to cells in serum-containing medium dropwise. At 24 h after transfection, the transfection medium was replaced with the refresh cell growth medium.

**TABLE 1:** The quantitative primer sequences and siRNA sequences.

**Primer sequence (5′-3′)**	
METTL3	F: TTG​TCT​CCA​ACC​TTC​CGT​AGT
	R: CCA​GAT​CAG​AGA​GGT​GGT​GTA​G
	METTL14	F: GAA​CAC​AGA​GCT​TAA​ATC​CCC​A
R: TGT​CAG​CTA​AAC​CTA​CAT​CCC​TG	
WTAP		F: TTC​CCA​AGA​AGG​TTC​GAT​TG
	R: TGC​AGA​CTC​CTG​CTG​TTG​TT
	VIRMA	F: AGT​CCG​AGT​CAT​ACC​CCC​AG
R: ACC​TAT​CGA​AAA​CAG​GGG​CA	
FTO		F: AAC​GAG​AGC​GCG​AAG​CTA​AG
	R: CTC​CTC​AGA​TAC​ACT​GCT​GG
	ALKBH5	F: TTC​GGC​TGC​AAG​TTC​CAG​TTC
R: TTG​ATG​TCC​TGA​GGC​CGT​ATG	
JAK2		F: TGT​CTT​ACC​TCT​TTG​CTC​AGT​GGC​G
	R: CAA​TGA​CAT​TTT​CTC​GCT​CGA​CAG​C
	IGF2BP1	F: AAG​ACC​TTA​CCC​TTT​ACA​ACC​C
R: GCA​GCC​ACA​TCA​TTC​TCA​TAG	
β-actin		F: CTC​CAT​CCT​GGC​CTC​GCT​GT
	R: GCT​GTC​ACC​TTC​ACC​GTT​CC
	**siRNA sequence (5′-3′)**	
IGF2BP1#1	AUG​AAA​CAU​AAC​UUU​CUU​GUU
IGF2BP1#2	UUA​AUC​UAC​AGA​UAC​UGA​CAG
IGF2BP1#3	AGC​AUU​UUU​UUU​AAG​UCA​CUC
METTL3#1	GCA​CUU​GGA​UCU​ACG​GAA​U
METTL3#2	CGACUACAGUGCUGCCUU
METTL3#3	GCA​AGU​AUG​UUC​ACU​AUG​AAA

### Cell Counting Kit-8 (CCK-8) Assay

Cell proliferative ability was evaluated through CCK-8 assay (MedChemExpress, Monmouth Junction, NJ, United States). Briefly, transfected or non-transfected cells (4,000 cells per well) were plated into the 96-well plates and cultured in complete medium. At the indicated time points after ox-LDL treatment, 10 µl of CCK-8 solution was added to each well. Three hours later, the optical density (OD) values were determined at the wavelength of 450 nm.

### Transwell Migration Assay

The migratory ability of HUVECs was assessed through the Transwell migration assay. Briefly, HUVECs (50,000 cells) in serum-free medium were plated into the upper chamber of 24-well transwell plates (8-μm filter membranes, Costar, Cambridge, MA, United States) and complete medium was added into the lower chamber. After 24 h of incubation, cells migrated to the bottom surface of the membranes were fixed with 4% paraformaldehyde, stained with crystal violet solution (Sigma-Aldrich), and imaged using a microscope. The average number of migrated cells was examined in five random fields.

### Tube Formation Assay

HUVECs (10,000 cells per well) were seeded into 96-well plates coated with Matrigel (Corning, New York, NY, United States). At 12 h after ox-LDL treatment, cells were imaged and tube formation ability was assessed by total tube length. Tube-like structures represent the formed endothelial cord that was connected at both ends. The total tube length was determined using the Image-Pro Plus software (Media Cybernetics, Inc., Houston, TX, United States).

### M6A Level Determination

The percentage of m6A methylation level in total RNA was quantified using the m6A RNA Methylation Assay Kit (Colorimetric) (cat. no. ab185912, Abcam, Cambridge, United Kingdom) according to the protocols of the manufacturer. Briefly, negative control, positive control, and our RNA samples were co-incubated with the Binding Solution at 37°C for 90 min. After being washed three times using the Diluted Wash Buffer, the Diluted Capture Antibody was added to each well. After 60 min of incubation at room temperature and 3 times of rinse, the samples were co-incubated with the Diluted Detection Antibody at room temperature for 30 min. Next, the Diluted Detection Antibody solution was removed from each well, and each well was washed 4 times with the Diluted Wash Buffer. Subsequently, the Diluted Enhancer Solution was added to each well for 30 min at room temperature. After being washed 5 times, each well was added with the Developer Solution. After 10 min of incubation away from light at room temperature, the enzyme reaction was stopped by the Stop Solution, and the absorbance was measured at 450 nm. The m6A percentage in total RNA was calculated using the following formula: m6A% = (Sample OD − NC OD) ÷ S)/(PC OD − NC OD) ÷ *p*) × 100%. NC: negative control; PC: positive control; S: the amount of input sample RNA; *p*: the amount of input positive control. Equal amounts of RNA samples were used.

### RT-qPCR Assay

RNA was isolated from HUVECs using Trizol reagent (Thermo Scientific, Waltham, MA, United States) referring to the manufacturer’s protocols. The synthesis reaction of the cDNA first strand was performed using M-MLV Reverse Transcriptase (Thermo Scientific). Equal amounts of RNA samples were used in reverse transcription reactions. Next, cDNA was amplified and quantified using SYBR Green PCR Master Mix (Thermo Scientific). The relative expression levels of target genes were calculated using the 2^-(target CT−reference CT)^ formula. β-actin functioned as the reference gene. The quantitative primer sequences were displayed in Table 1.

### Western Blot Assay

The whole-cell lysates were prepared using RIPA Lysis buffer (Solarbio Life Sciences Co., Ltd., Beijing, China) supplemented with protease and phosphatase inhibitors (Sigma-Aldrich). Next, the concentrations of proteins were determined using the Bio-Rad Bradford Protein Assay Kit (Bio-Rad Laboratories, Hercules, CA, United States). An equal amount of protein (30μg/lane) was separated through SDS-PAGE and transferred to nitrocellulose membranes (Millipore, Bedford, MA, United States). After the blockade of non-specific signals using 5% skim milk, the membranes were incubated with anti-METTL3 (1/1,000 dilution; cat. no. ab195352, Abcam), anti-VEGFA (1/1,000 dilution; cat. no. ab214424, Abcam), anti-JAK2 (1/1,000 dilution; cat. no. #3230, Cell Signaling Technology, Danvers, Massachusetts, United States), anti-p-STAT3 (tyr705) (1/1,000 dilution; cat. no. #9131, Cell Signaling Technology), anti-STAT3 (1/1,000 dilution; cat. no.#4904, Cell Signaling Technology), anti-IGF2BP1 (1/1,000 dilution; cat. no. #8482, Cell Signaling Technology), or anti-β-actin (1/2000 dilution; cat. no. ab8227, Abcam) for more than 12 h at 4˚C. Next, the membranes were incubated with secondary antibody conjugated with horseradish peroxidase (HRP) for 1.5 h at room temperature. Finally, immunoreactive protein bands were visualized using the Pierce ECL Western Blotting Substrate (Thermo Scientific). Target proteins were quantified based on the greyscale values using the Image-Pro Plus software (Media Cybernetics, Inc.) with β-actin as the internal control. Equal amounts of protein samples were used.

### ELISA Assay

VEGF levels in cell supernatants of one million HUVECs or equal amounts of mouse AS lesion tissue homogenates were determined using a human or mouse VEGF ELISA kit (Abcam) according to the instructions of the manufacturer, respectively.

### Bioinformatics Analysis

Venn analysis was carried out using the jvenn website to screen out common genes between/among gene lists (Bardou et al., 2014). KEGG and GO enrichment analysis was performed using the DAVID database to identify the KEGG pathways and GO terms that are statistically significantly associated with the input gene lists (Huang et al., 2009a; Huang et al., 2009b). And, mRNAs that could bind with METTL3 and m6A readers (YTHDF1, YTHDC1, IGF2BP1, IGF2BP2, IGF2BP3, HNRNPC, and HNRNPA2B1) with a possibility to interact with JAK2 mRNA (Genbank accession number: NM_004972.4) were predicted by the starBase database (Li et al., 2014). The potential m6A methylation sites in JAK2 mRNA (Genbank accession number: NM_004972.4) were predicted by the SRAMP predictor (Zhou et al., 2016).

### mRNA Stability Detection

At 48 h after transfection, HUVECs were co-incubated with actinomycin D (ActD, 5 μg/ml, Sigma-Aldrich). At 0, 2, 4, or 6 h after ActD treatment, total RNA was extracted from HUVECs and JAK2 mRNA level was measured through RT-qPCR assay.

### RIP Assay

RIP assay was conducted using Magna RIP RNA-Binding Protein Immunoprecipitation Kit (Millipore) and the antibody against IGF2BP1 (cat. no. ab184305, Abcam) or IgG (cat. no. ab172730, Abcam) in HUVECs. Equal amounts of cell samples were used. Briefly, the complexes of magnetic beads and antibodies were co-incubated with the cell lysates for more than 12 h at 4°C. Next, RNA was eluted, extracted, and purified. JAK2 mRNA level was measured through RT-qPCR assay.

### MeRIP Assay

The m6A modification level in JAK2 mRNA was examined through Magna MeRIP m6A Kit (Millipore) following the protocols of the manufacturer. After extracted, purified, and fragmented, total RNA was co-incubated with magnetic beads conjugated with m6A antibody. Next, methylated RNA was eluted and purified. JAK2 mRNA level was examined through RT-qPCR assay. Equal amounts of total RNA samples were used.

### RNA Pull-Down Assay

RNA pull-down assay was performed through the Pierce Magnetic RNA-Protein Pull-Down Kit (Thermo Scientific) to investigate whether IGF2BP1 protein could bind with JAK2 mRNA via putative binding sites. Briefly, cell lysates were prepared using Pierce IP Lysis Buffer (Thermo Scientific). Next, cell lysates containing equal amounts of proteins (60 μg) were co-incubated with streptavidin-conjugated magnetic beads and biotin-labeled wild or mutant JAK2 coding region (CDS) (CDS-wt or CDS-mut) probe. Next, the protein was eluted from the magnetic beads, and the IGF2BP1 protein level was detected through western blot assay.

### Luciferase Reporter Assay

The wild or mutant JAK2 CDS sequence was constructed into the pGL3-Basic plasmid by GeneCreate Biological Engineering Co., Ltd., and generated recombinant plasmid was named as JAK2-wt or JAK2-mut reporter, respectively. Next, the recombinant reporter and pRL-TK Renilla luciferase plasmid were co-transfected into HUVECs together with overexpressing plasmid or siRNA. At 48 h after transfection, luciferase activities were measured through a Dual-Luciferase Reporter Assay kit (Promega, Madison, WI, United States). Renilla luciferase activity functioned as the internal control to normalize the firefly luciferase activity.

### Animal Experiments

All animal procedures were approved by the Ethics Committee of our hospital and conducted with the Guide for the Care and Use of Laboratory Animals. Eight-week-old male ApoE^−/−^ mice (n = 10) were purchased from Beijing HFK Bioscience Co., Ltd. (Beijing, China) and housed in a specific pathogen-free environment with a 12-h light/dark cycle. All mice had free access to fresh water and feed. The adeno-associated viruses (AAV) that could silence METTL3 (sh-METTL3) and the negative control adeno-associated viruses (sh-NC) were obtained from WZ Biosciences Inc. (Jinan, China). ApoE^−/−^ mice were randomly divided into AS + sh-NC and AS + sh-METTL3 groups. Each group contains five mice. Mice were fed with the standard diet for 1 week to acclimatize. After 1 week of acclimation, mice were challenged with a high-fat and high-cholesterol feed H10540 (Beijing HFK BIOSCIENCE Co., Ltd., Beijing, China). The formula of the H10540 feed was shown in Supplementary File S1. After 8 weeks of HFD feeding, sh-NC or sh-METTL3 adeno-associated virus serotype 9 (AAV9, 10^12^ viral genome copies per mouse) were respectively delivered into mice in AS + sh-NC or AS + sh-METTL3 group through tail vein injection. At 14 weeks after HDF feeding, mice fasted overnight. Next, blood samples were collected from mouse orbital venous plexus and the plasma levels of total cholesterol (TC), triglyceride (TG), low-density lipoprotein-cholesterol (LDL-C), and high-density lipoprotein-cholesterol (HDL-C) were determined using a corresponding colorimetric assay kit (Elabscience Co., Ltd., Wuhan, China), respectively. Next, mice hearts were exposed and sequentially infused with normal saline. The aorta was isolated, fixed, and stained with Oil Red O. Next, stained aortas were imaged using a digital camera and analyzed using Image-Pro Plus software (Media Cybernetics, Inc., Houston, TX, United States). Plaque area (%) in the aorta was calculated as follows: plaque area (%) = Oil red O-positive area/total area × 100%. Also, the sections of the aortic valve were stained with Oil Red O as previously described (Jian et al., 2020).

### Immunofluorescence (IF) Assay

p-STAT3 level in AS lesions were measured by IF assay. Briefly, aortic arch tissue sections were blocked with 3% BSA after the routine treatment. Next, the sections were sequentially incubated with the first primary antibody (anti-CD31, 4°C, overnight) and corresponding HRP-conjugated secondary antibody (room temperature, 50 min). Then, the CY3-TSA solution was added to the sections. After 10 min of incubation at room temperature in the dark and antigen retrieval treatment, the sections were sequentially incubated with the second primary antibody (anti-p-STAT3, 4°C, overnight), matching HRP-labelled secondary antibody (room temperature, 50 min), and FITC-TSA (room temperature, 10 min, dark place). Next, the cell nuclei were counterstained with the DAPI solution. After the treatment of quenching spontaneous fluorescence and mounting, the sections were imaged using a fluorescent microscope, and the stained area was measured using Image Pro Plus software (Media Cybernetics, Inc). The percent of p-STAT3^+^ cells was determined as follows: p-STAT3+ cell percentage = the number of p-STAT3^+^ cells/the number of total cells × 100%.

### Chick Embryo Chorioallantoic Membrane (CAM) Assay

Fertilized chicken eggs (7-day-old) were used in the CAM assay. The medium isolated from HUVECs with or without siRNA and plasmid, or/and ox-LDL treatment was applied on the CAM region, restricted by a rubber ring. Three days later, the area inside the ring was imaged and the angiogenetic ability was assessed through the vascular density. The density of blood vessels was examined using the Image-Pro Plus software (Media Cybernetics, Inc.).

### Statistical Analysis

Data analysis was performed using GraphPad Prism software (Version 7, GraphPad Software, Inc., San Diego, CA, United States) with the outcomes presenting as mean ± standard deviation. The difference between groups was compared using Student’s t-test, while the difference among groups was analyzed using one-way ANOVA (Tukey’s post-hoc test) or two-way ANOVA (Sidak post-hoc test). The difference was regarded significantly different when the *p*-value was less than 0.05.

## Results

### METTL3 was Highly Expressed in Dysregulated HUVECs Induced by ox-LDL

**FIGURE 1:**
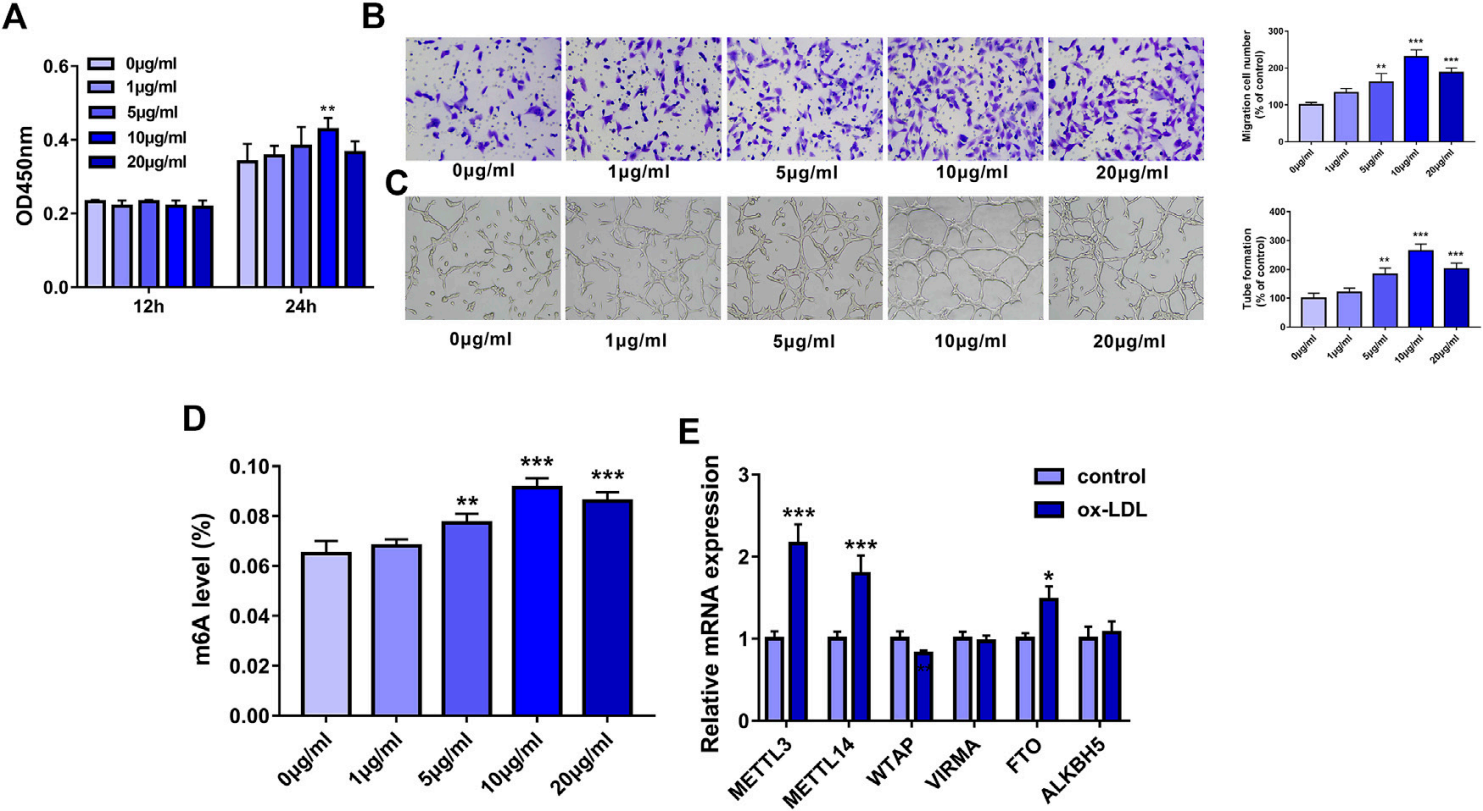
METTL3 was highly expressed in ox-LDL-treated HUVECs. **(A–D)** HUVECs were treated with different concentrations of ox-LDL (0, 1, 5, 10, or 20 μg/ml). **(A)** Cell proliferative activity was examined through CCK-8 assay at 12 h or 24 h after ox-LDL exposure. **(B)** Cell migratory ability was analyzed using Transwell migration assay at 24 h after ox-LDL treatment. **(C)** Tube formation capacity was assessed by tube formation assay at 12 h after ox-LDL stimulation. **(D)** The global m6A level in total RNA was measured using a commercial kit at 24 h after ox-LDL stimulation. **(E)** At 24 h after ox-LDL treatment, mRNA levels of METTL3, METTL14, WTAP, VIRMA, FTO, and ALKBH5 were measured through RT-qPCR assay. Three independent experiments were performed for all *in vitro* assays. *p < 0.05, **p < 0.01, ***p < 0.001.

Firstly, our study demonstrated that cell proliferative ability was notably increased in HUVECs following a 24-h exposure to 10 μg/ml of ox-LDL (Figure 1A). Also, the Transwell migration assay showed that ox-LDL concentration-dependently improved the migratory potential of HUVECs, reaching the peak value at the concentration of 10 μg/ml (Figure 1B). Moreover, ox-LDL stimulation led to the marked elevation of tube formation capacity in a dose-dependent manner in HUVECs, reaching a maximum of 10 μg/ml (Figure 1C). Recent mass spectrometry analysis showed that RNA m6A methylation modification levels and the expression levels of some m6A regulators were dysregulated in AS lesions (Quiles-Jiménez et al., 2020). Moreover, several m6A regulators have been reported to be involved in the regulation of AS progression (Chien, et al., 2021; Jian et al., 2020). Thus, the global m6A level in total RNA was measured in HUVEC stimulated with or without ox-LDL. Results showed that the whole m6A level in total RNA was markedly increased in HUVECs exposed to ox-LDL (5, 10, or 20 μg/ml) with the highest level at the dose of 10 μg/ml (Figure 1D). Thus, 10 μg/ml of ox-LDL was used to induce the dysfunction of HUVECs in the following experiments. Next, the expression levels of four m6A writers (METTL3, METTL14, WTAP, and VIRMA) (Jiang, et al., 2021) and two m6A erasers (FTO and ALKBH5) (He and He, 2021) were examined in HUVECs treated with or without ox-LDL. The outcomes showed that METTL3, METTL14, and FTO expression levels were noticeably upregulated in HUVECs exposed to ox-LDL compared to the control group (Figure 1E). Of these upregulated m6A enzymes, METTL3 had the highest upregulation ratio. Hence, METTL3 was picked out for further investigations.

### METTL3 Knockdown Inhibited Cell Proliferation, Migration, Tube Formation, and VEGF Expression in ox-LDL-Treated HUVECs

**FIGURE 2:**
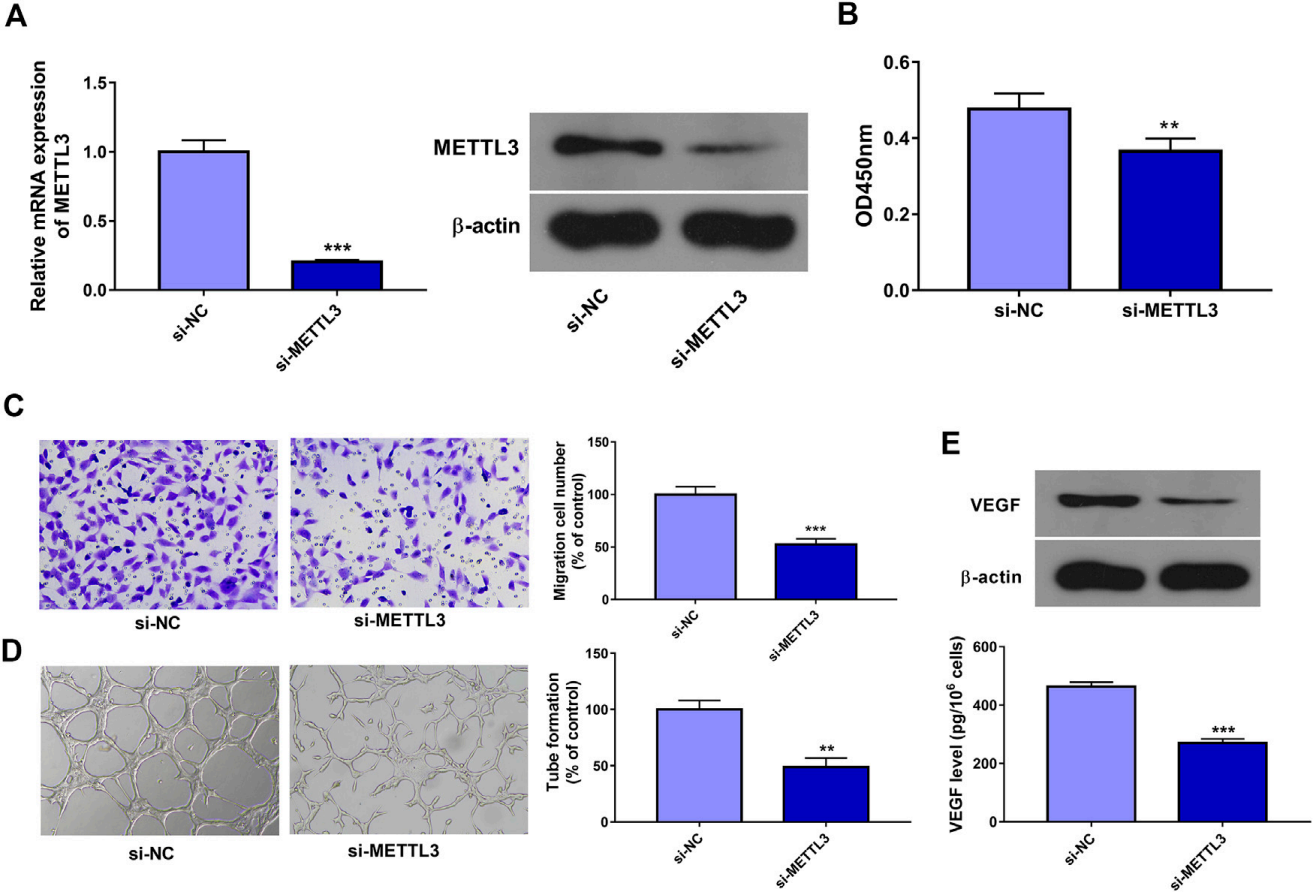
METTL3 knockdown inhibited cell proliferation, migration, tube formation, and VEGF expression/secretion in ox-LDL-treated HUVECs. **(A)** HUVECs were transfected with si-NC or si-METTL3. Forty-eight hours later, METTL3 mRNA and protein levels were measured through RT-qPCR and western blot assays, respectively. **(B–E)** HUVECs transfected with si-NC or si-METTL3 were stimulated with 10 μg/ml of ox-LDL. **(B, C)** At 24 h after ox-LDL treatment, cell proliferative **(B)** and migratory **(C)** abilities were measured through CCK-8 and Transwell migration assays, respectively. **(D)** At 12 h after ox-LDL exposure, tube formation capacity was assessed by tube formation assay. **(E)** VEGF protein expression and secretion levels were determined through western blot and ELISA assays at 24 h after ox-LDL treatment, respectively. Three independent experiments were performed for all *in vitro* assays. **p < 0.01, ***p < 0.001.

In this text, 3 siRNAs targeting METTL3 (si-METTL3#1, si-METTL3#2, and si-METTL3#3) were designed and synthesized. Transfection efficiency analysis revealed that the introduction of si-METTL3#1, si-METTL3#2, or si-METTL3#3 could notably decrease METTL3 expression in HUVECs (Data not presented). Hence, the equal ratio mixture of si-METTL3#1, si-METTL3#2, and si-METTL3#3, named si-METTL3, was used in the following experiments. Next, knockdown efficiency analysis revealed that the transfection of si-METTL3 led to the notable reduction of METTL3 mRNA and protein levels in HUVECs compared to the si-NC group (Figure 2A). CCK-8 assay showed that METTL3 knockdown markedly hindered cell proliferation in HUVECs treated with ox-LDL (Figure 2B). Transwell migration assay revealed that METTL3 depletion triggered the noticeable reduction of cell migratory ability in HUVECs stimulated with ox-LDL (Figure 2C). Moreover, METTL3 loss conspicuously weakened the tube formation potential of ox-LDL-exposed HUVECs (Figure 2D). Additionally, METTL3 knockdown led to the conspicuous decrease of VEGF expression and secretion levels in HUVECs treated with ox-LDL relative to the si-NC group (Figure 2E).

### METTL3 Knockdown Suppressed the JAK2/STAT3 Signaling Pathway by Reducing JAK2 Expression and mRNA Stability in an m6A Dependent Manner in HUVECs

**FIGURE 3:**
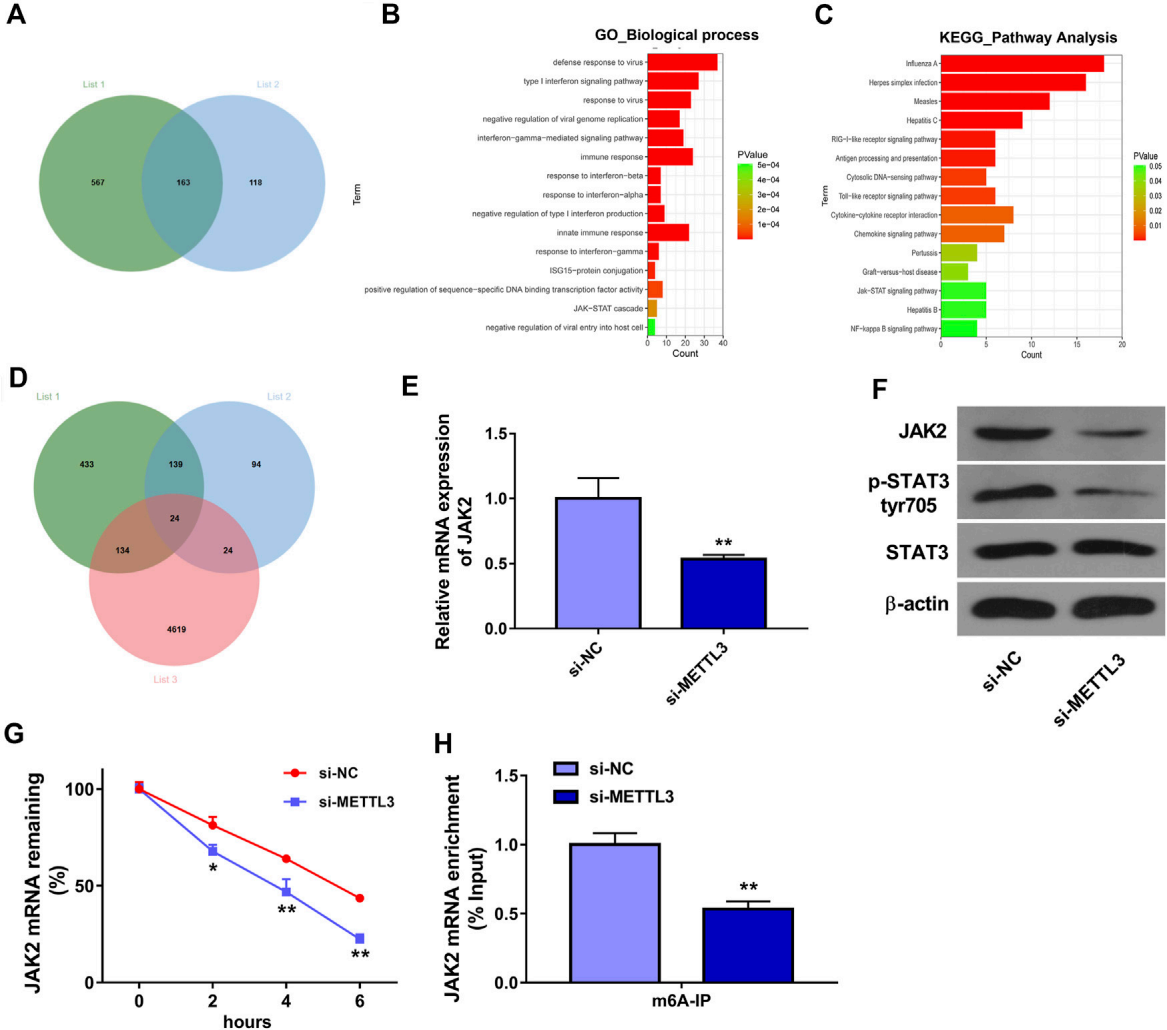
METTL3 positively regulated JAK2/STAT3 pathway by influencing the stability of JAK2 mRNA in an m6A-dependent manner. **(A)** Venn analysis for genes in Supplementary Tables S1, S2. **(B)** The top 15 biological process terms enriched by the common genes in Supplementary Tables S1, S2. **(C)** The top 15 KEGG pathways enriched by the common genes in Supplementary Tables S1, S2. **(D)** Venn analysis for genes in Supplementary Tables S1, S2, and S4. **(E–H)** HUVECs were transfected with si-NC or si-METTL3 for 48 h **(E)** JAK2 mRNA level was measured through RT-qPCR assay. **(F)** Protein levels of JAK2, p-STAT3, and STAT3 were determined by western blot assay. **(G)** JAK2 mRNA stability was tested by ActD assay. **(H)** The m6A modification level in JAK2 mRNA was examined through MeRIP and RT-qPCR assay. Three independent experiments were performed for all *in vitro* assays. *p < 0.05, **p < 0.01.

To further screen out genes that could be regulated by METTL3 in an m6A-dependent manner, we downloaded Table S6 and Table S7 from Wang’s project (Wang et al., 2020b). Table S6 in Wang’s project (Wang et al., 2020b) presented the mRNAs with the notable difference (|log_2_FoldChange| > 1 and *p* < 0.05) in m6A methylation levels in METTL3-deficient HUVECs (METLL3 loss group) compared to the wild-type group (control group). Table S7 in Wang’s project (Wang et al., 2020b) listed the differentially expressed genes (|log_2_FoldChange| > 0.27 and *p* < 0.05) in METTL3-depleted HUVECs relative to the control group. It is well known to us that METTL3 is an m6A methyltransferase. Hence, genes with a notable reduction in the mRNA m6A methylation levels in the METLL3 loss group versus the control group were screened out from Table S6 in Wang’s project (Wang et al., 2020b), and these genes were shown in Supplementary Table S1 (list 1). To identify genes that could be positively regulated by METTL3, the downregulated genes (|log_2_FoldChange| > 1 and *p* < 0.05) in the METLL3 loss group versus the control group were screened out from Table S7 in Wang’s project (Wang et al., 2020b) and presented in Supplementary Table S2 (list 2). Combined analysis for data in lists one and two suggested that METTL3 might catalyze the mRNA m6A methylation of 163 common genes to positively regulate the expression of these genes (Figure 3A). These 163 common genes were shown in Supplementary Table S3. GO enrichment analysis revealed that these 163 genes were significantly enriched in multiple biological processes including immune responses and JAK-STAT cascade (Figure 3B). KEGG enrichment analysis showed that these 163 genes mainly participated in the regulation of RIG-I-like and Toll-like receptor signaling pathways, cytokine-cytokine receptor interaction, and JAK-STAT signaling pathway (Figure 3C). Additionally, mRNAs that could bind with METTL3 were predicted by the starBase database and the prediction results were presented in Supplementary Table S4 (list 3). Venn analysis showed that there were 24 common genes in lists 1, 2, and 3 (Figure 3D and Supplementary Table S5). These 24 genes might directly interact with METTL3 and be positively regulated by METTL3 in an m6A dependent manner. Among these 24 genes, JAK2 was selected for further investigation considering its close link with the JAK-STAT signaling pathway. Moreover, JAK2/STAT3 signaling cascade has been reported to be involved in the regulation of AS process (Chithra et al., 2017; Yang et al., 2020). Thus, the effect of METTL3 knockdown on the JAK2/STAT3 pathway was examined in HUVECs. The outcomes presented that METTL3 depletion led to the noticeable reduction of JAK2 expression at mRNA (Figure 3E) and protein (Figure 3F) levels and p-STAT3 protein level (Figure 3F) in HUVECs. That was to say, METTL3 knockdown inhibited the JAK2/STAT3 signaling pathway in HUVECs. Additionally, our data revealed that METTL3 loss markedly reduced the stability of JAK2 mRNA in HUVECs (Figure 3G). Furthermore, the MeRIP assay demonstrated that METTL3 depletion triggered the notable reduction of m6A level in JAK2 mRNA in HUVECs (Figure 3H). In summary, these data showed that METTL3 knockdown suppressed JAK2/STAT3 signaling pathway by reducing JAK2 expression and mRNA stability in an m6A dependent manner. Additionally, western blot assay demonstrated that JAK2 protein expression level and p-STAT3 level were notably increased in HUVECs after ox-LDL treatment (Supplementary Figure).

### IGF2BP1 Positively Regulated JAK2 Expression and mRNA Stability by Directly Binding to an m6A Site Within the CDS of JAK2 mRNA, and METTL3 Knockdown Impaired the Interaction of IGF2BP1 and JAK2 mRNA in HUVECs

**FIGURE 4:**
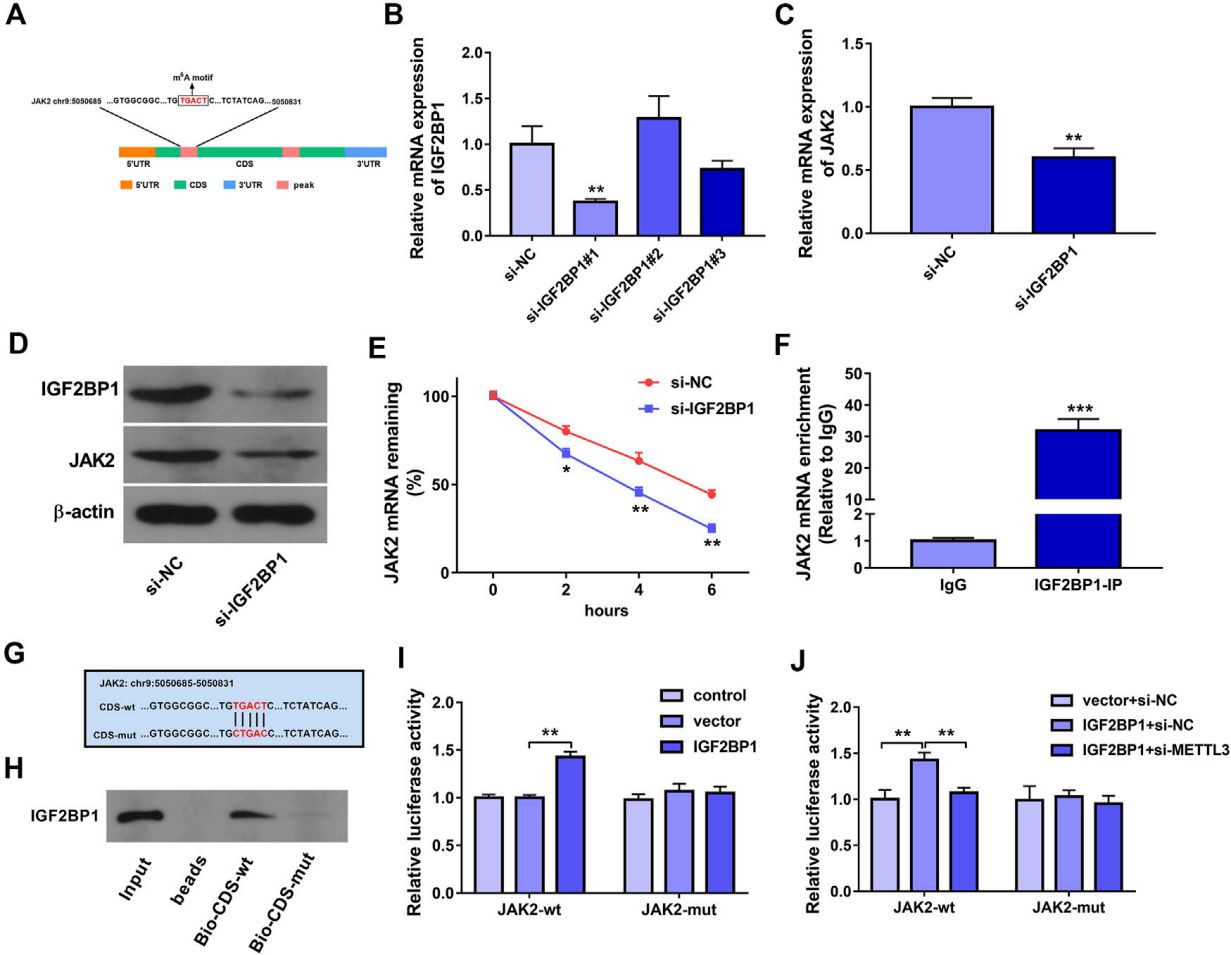
IGF2BP1 positively regulated JAK2 expression and mRNA stability by directly binding to an m6A site within the CDS of JAK2 mRNA, and METTL3 knockdown impaired the interaction of IGF2BP1 and JAK2 mRNA in HUVECs. **(A)** The location of this m6A methylation motif in JAK2 mRNA. **(B)** HUVECs were transfected with si-NC, si-IGF2BP1#1, si-IGF2BP1#2, or si-IGF2BP1#3. Forty-eight hours later, IGF2BP1 mRNA level was measured through RT-qPCR assay. **(C–E)** HUVECs were transfected with si-NC or si-IGF2BP1#1 for 48 h **(C)** JAK2 mRNA level was measured through RT-qPCR assay. **(D)** Protein levels of IGF2BP1 and JAK2 were measured by western blot assay. **(E)** The stability of JAK2 mRNA was examined by ActD assay. **(F)** RIP assay was performed in HUVECs using the antibody against IgG or IGF2BP1. JAK2 mRNA level in IgG or IGF2BP1 immunoprecipitation complex was measured by RT-qPCR assay. **(G)** The wild or mutant m6A methylation site in JAK2 mRNA. **(H)** RNA pull-down assay was conducted in HUVECs using biotin-labeled JAK2 CDS-wt or CDS-mut probe. IGF2BP1 protein level pulled down by biotin-labeled probe was measured through western blot assay. **(I, J)** Luciferase activities were measured at 48 h after transfection. Three independent experiments were performed for all *in vitro* assays. *p < 0.05, **p < 0.01, ***p < 0.001.

It has been reported that m6A readers play decisive roles in governing the fate of methylated mRNAs (Maity and Das, 2016; Robinson et al., 2019). Hence, m6A readers that could directly interact with JAK2 mRNA were predicted through the starBase database. Combined with the data in Table S6 in Wang’s project (Wang et al., 2020b), we found that there was a potential IGF2BP1 binding site in one of METTL3-influenced JAK2 m6A peaks. Additionally, SRAMP prediction analysis revealed that there was a potential m6A methylation site in the above METTL3-influenced JAK2 m6A peak. The location of this m6A methylation motif in JAK2 mRNA was presented in Figure 4A. Moreover, previous studies demonstrated that IGF2BPs could positively regulate gene expression by increasing the stability of target mRNAs in an m6A-dependent mode (Huang et al., 2018). Thus, we supposed that m6A reader IGF2BP1 might be involved in mediating the regulatory effects of METTL3 on JAK2 in an m6A-dependent manner. To validate the hypothesis, siRNAs targeting IGF2BP1 (si-IGF2BP1#1, si-IGF2BP1#2, and si-IGF2BP1#3) and si-NC were synthesized. The RT-qPCR assay showed that the transfection of si-IGF2BP1#1 led to the marked reduction of IGF2BP1 mRNA level in HUVECs (Figure 4B). Western blot assay further validated that IGF2BP1 protein level was notably decreased in HUVECs transfected with si-IGF2BP1#1 than that in si-NC-transfected cells (Figure 4D). Thus, si-IGF2BP1#1 was used in the following experiments. Moreover, RT-qPCR and western blot assays demonstrated that IGF2BP1 knockdown led to the notable reduction of JAK2 expression at mRNA and protein levels in HUVECs (Figures 4C,D). Additionally, IGF2BP1 loss triggered the noticeable reduction of JAK2 mRNA stability in HUVECs (Figure 4E). Furthermore, the RIP assay disclosed that JAK2 could be significantly enriched by IGF2BP1 antibody in HUVECs (Figure 4F), suggesting that IGF2BP1 could interact with JAK2 mRNA. To further explore whether IGF2BP1 could directly bind with JAK2 mRNA through the putative m6A motif (TGACT), biotin-labeled JAK2 CDS-wt and CDS-mut probes were synthesized. The mutant site was presented in Figure 4G. RNA pull-down assay further demonstrated that IGF2BP1 could be pulled down by biotin-labeled JAK2 CDS-wt probe, but not by CDS-mut probe (Figure 4H). Additionally, JAK2-wt or JAK2-mut reporter containing wild or mutant IGF2BP1 binding site was constructed, respectively. Luciferase reporter assay revealed that IGF2BP1 overexpression markedly increased the luciferase activity of JAK2-wt reporter, but did not influence the luciferase activity of JAK2-mut reporter (Figure 4I). Furthermore, METTL3 knockdown inhibited the increase of JAK2-wt reporter luciferase activity mediated by IGF2BP1 (Figure 4J), suggesting that METTL3 loss could weaken the interaction between JAK2 mRNA and IGF2BP1.

### METTL3 Exerted its Functions Through Regulating JAK2 Expression and JAK2/STAT3 Pathway

**FIGURE 5:**
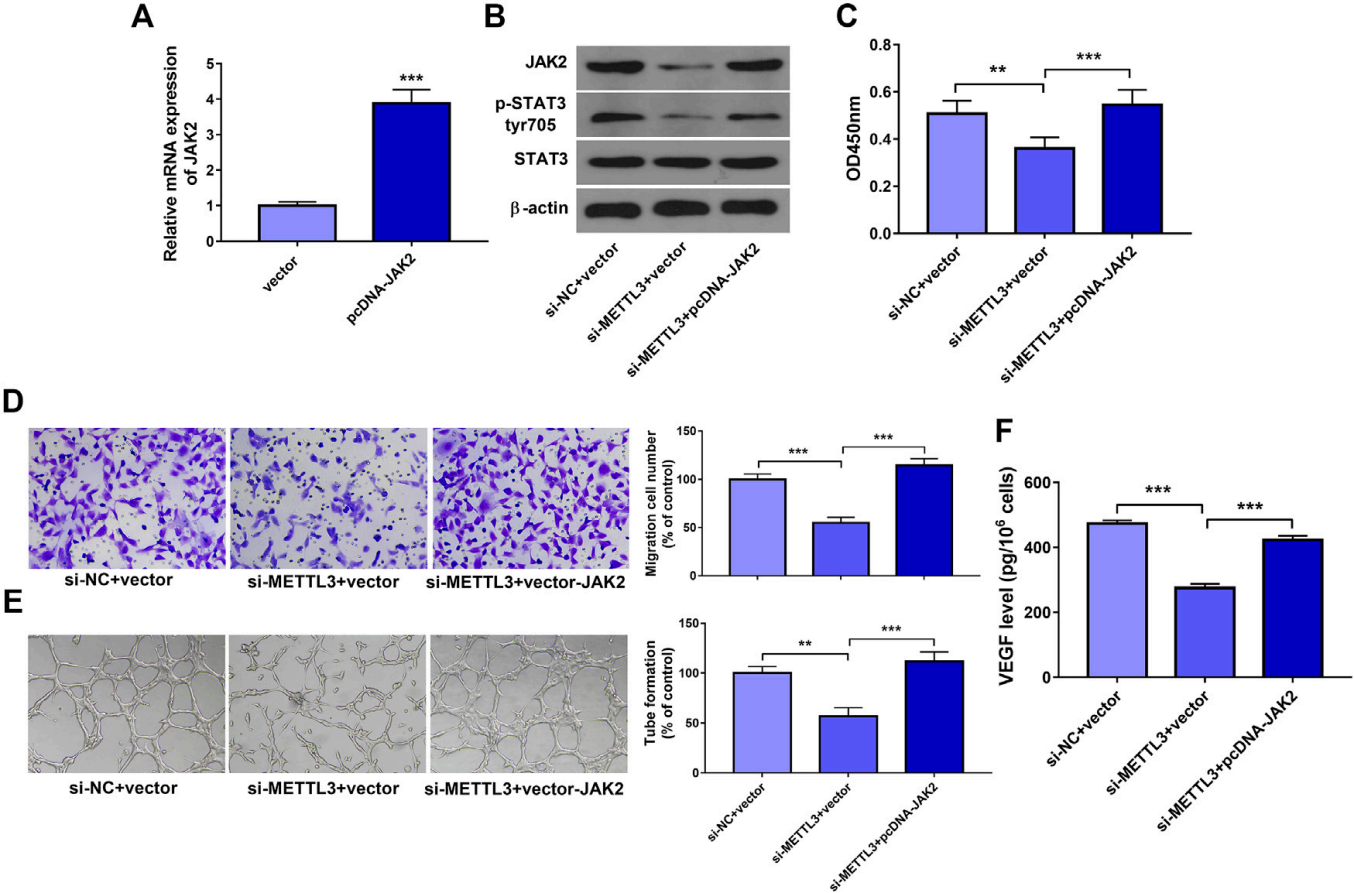
METTL3 exerted its functions by regulating JAK2 expression and JAK2/STAT3 pathway. **(A)** HUVECs were transfected with pcDNA-JAK2 plasmid or empty vector. Forty-eight hours later, JAK2 mRNA level was measured by RT-qPCR assay. (B–F) HUVECs were co-transfected with si-NC + pcDNA3.1, si-METTL3+pcDNA3.1, or si-METTL3+pcDNA-JAK2 for 24 h and then stimulated with ox-LDL. At 24 h after ox-LDL treatment, JAK2, p-STAT3, and STAT3 protein levels **(B)**, cell proliferative **(C)** and migratory **(D)** capacities, and VEGF secretion level **(F)** were measured. **(E)** At 12 h after ox-LDL treatment, tube formation patterns were measured. Three independent experiments were performed for all *in vitro* assays. **p < 0.01, ***p < 0.001.

To further explore whether METTL3 could exert its functions by regulating JAK2 expression and JAK2/STAT3 pathway, JAK2 overexpression plasmid pcDNA-JAK2 was constructed. Transfection efficiency analysis revealed that the introduction of pcDNA-JAK2 plasmid led to the notable increase of JAK2 mRNA level in HUVECs (Figure 5A). Moreover, JAK2 overexpression weakened METTL3 loss-mediated downregulation of p-STAT3 level in HUVECs (Figure 5B). Additionally, enforced expression of JAK2 abrogated the detrimental effects of METLL3 depletion on cell proliferation (Figure 5C), migration (Figure 5D), tube formation (Figure 5E), and VEGF secretion (Figure 5F) in HUVECs. In conclusion, these data revealed that METLL3 knockdown hindered cell proliferation, migration, and angiogenesis by downregulating JAK2 expression and inhibiting JAK/STAT pathway in HUVECs.

### METTL3 Knockdown Hampered AS Progression by Inhibiting JAK2/STAT3 Pathway *In Vivo*

**FIGURE 6:**
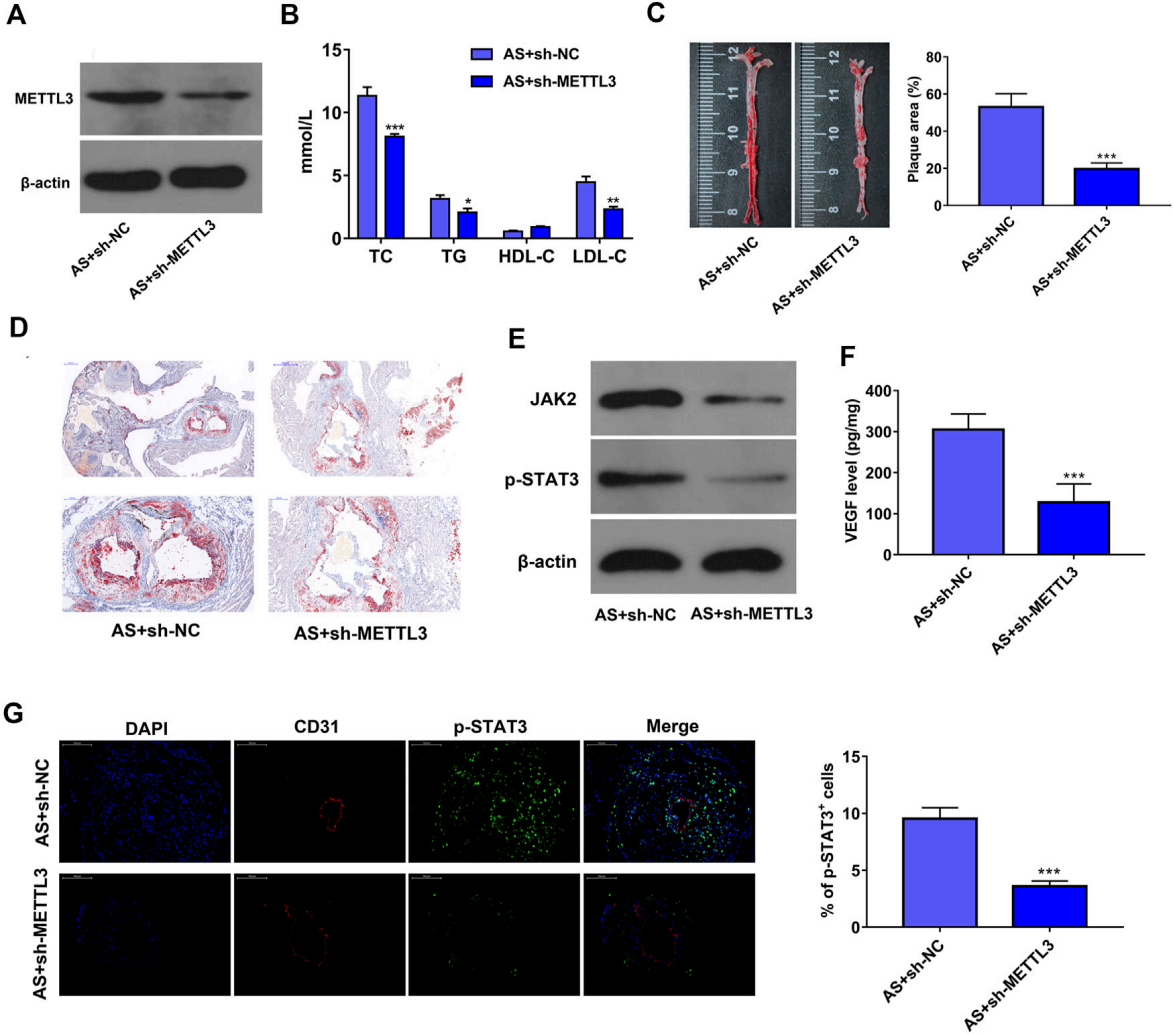
METTL3 knockdown hampered AS progression by inhibiting JAK2/STAT3 pathway *in vivo*. **(A)** Protein level of METTL3 in AS lesions was measured by western blot assay (n = 5). **(B)** The plasma levels of TC, TG, HDL-C, and LDL-C were measured through corresponding kits (n = 5). **(C)** Representative images of the total aorta stained with Oil Red O, and the estimation of AS lesion area (n = 5). **(D)** Representative images of aortic valve sections stained with Oil Red O (n = 5). **(E)** Protein levels of JAK2 and p-STAT3 in AS lesions were measured by western blot assay (n = 5). **(F)** VEGF secretion level in the lesions was examined through ELISA assay (n = 5). **(G)** Protein levels of p-STAT3 in AS lesions were measured by IF assay (magnification: ×200, n = 3). Previously frozen mice samples were used to measure the level of JAK2 and p-STAT3. *p < 0.05, **p < 0.01. ***p < 0.001.

Firstly, we demonstrated that METTL3 level was notably reduced in AS lesions of sh-METTL3 mice compared to the sh-NC group (Figure 6A). It is generally known that dyslipidemia and lipid deposition is a common feature of AS (Teramoto et al., 2013). Hence, the plasma lipoprotein levels were measured in mice at the end of the experiments. Our data revealed that METTL3 knockdown led to the notable reduction of TC, TG, and LDL-C plasma levels and marked increase of HDL-C plasma level in AS mouse models (Figure 6B), suggesting that METTL3 loss noticeably inhibited lipid accumulation in the blood of AS mice. Moreover, reduced AS plaque deposition was observed in the aortic tissues of mice in the sh-METTL3 group compared to the sh-NC group (Figures 6C,D), suggesting that METTL3 depletion hindered AS plaque formation *in vivo*. Additionally, METTL3 loss triggered the notable reduction of JAK2 and p-STAT3 protein expression, and VEGF secretion level in AS lesions (Figures 6E,F). Furthermore, IF assay showed that the protein levels of p-STAT3 were notably reduced in the AS lesions of the AS + sh-METTL3 group mice compared to the AS + sh-NC group (Figure 6G). These outcomes suggested that METTL3 knockdown hindered the development of AS by inactivating the JAK2/STAT3 pathway *in vivo*.

### METTL3 Knockdown Hindered ox-LDL-Induced Angiogenesis by Regulating JAK2 Expression *In Vivo*

**FIGURE 7:**
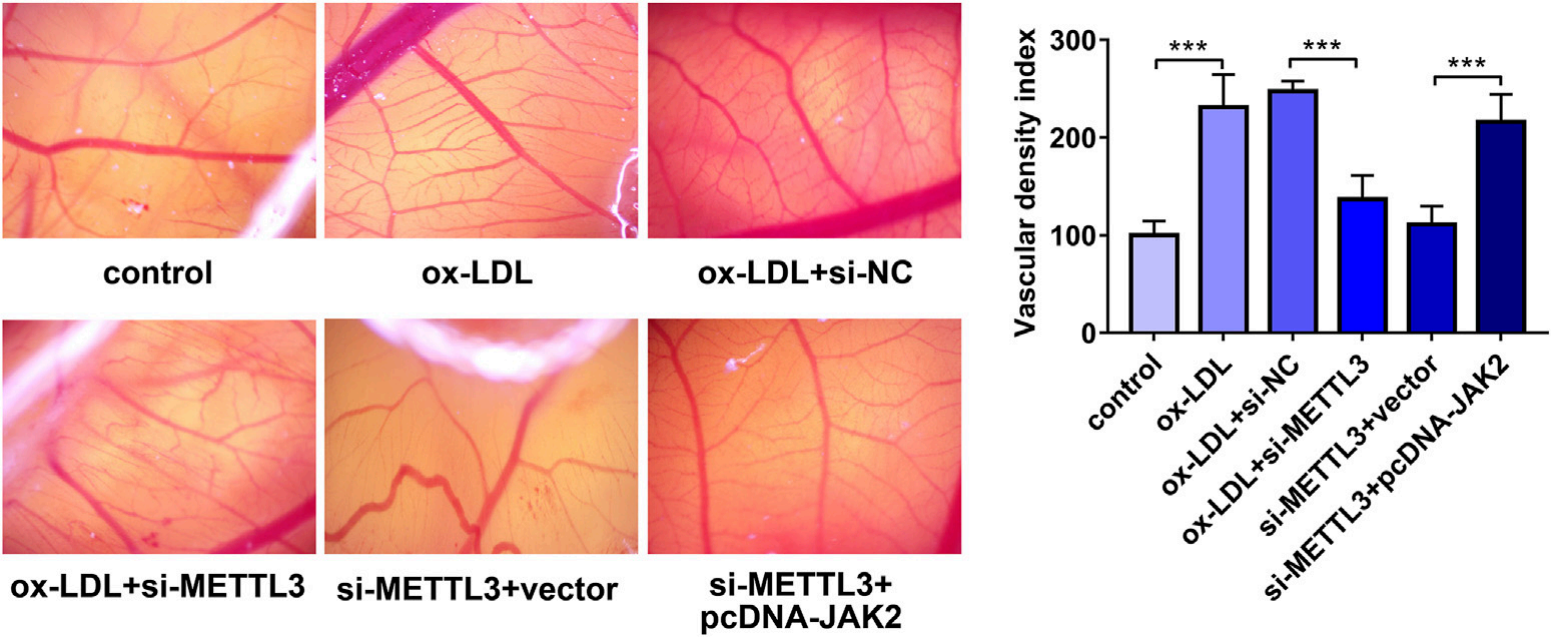
METTL3 knockdown hindered ox-LDL-induced angiogenesis by regulating JAK2 *in vivo*. HUVECs were transfected with si-NC, si-METTL3, si-METTL3+vector, or si-METTL3+pcDNA-JAK2. Twenty-four hours later, HUVECs were stimulated with ox-LDL (10 μg/ml) for an additional 24 h. Next, the conditioned medium was collected and added to the CAM (n = 5). Three days later, the angiogenetic ability was assessed. ***p < 0.001.

Next, a CAM assay was performed to further investigate the effect of METTL3 knockdown on angiogenesis *in vivo*. Results showed that ox-LDL stimulated angiogenesis and METTL3 knockdown inhibited ox-LDL-induced angiogenesis (Figure 7). Moreover, restoration experiments demonstrated that JAK2 overexpression notably alleviated the inhibitory effects of METTL3 loss on ox-LDL-induced angiogenesis *in vivo* (Figure 7).

## Discussion

In this project, our data revealed that METTL3 expression was noticeably increased in ox-LDL-induced dysregulated HUVECs. Moreover, METTL3 knockdown weakened cell proliferative, migratory, and tube formation capacities in ox-LDL-exposed HUVECs *in vitro* and hindered *in vivo* angiogenesis of developing embryos. It has been reported that the migration, proliferation, and capillary tube formation of endothelial cells are essential for angiogenesis, and intra-plaque angiogenesis can facilitate AS progression (Perrotta et al., 2019; Ucuzian and Greisler, 2007). Thus, we supposed that METTL3 loss might hinder AS process. As expected, our *in vivo* investigations demonstrated that METTL3 depletion reduced blood lipid burden and inhibited AS plaque formation in the AS mouse models. Consistent with our outcomes, a recent study also demonstrated that METTL3 functioned as a crucial player in the response to hemodynamic forces and atherogenic stimuli in endothelial cells and METTL3 knockdown prevented the development of AS in the *in vivo* AS model induced by partial carotid-artery ligation (Chien, et al., 2021).

Moreover, we further demonstrated that METTL3 positively regulated JAK2 expression and JAK2/STAT3 pathway in an m6A-dependent manner in HUVECs. Additionally, METTL3 knockdown hindered the development of AS *in vitro* and *in vivo* by inhibiting JAK2/STATA3 pathway. The positive regulatory effect of METTL3 on JAK2 expression and JAK2/STAT3 pathway also has been identified by a prior study, which demonstrated that METTL3 knockdown inhibited cell self-renewal and induced cell differentiation by reducing JAK2 expression and inactivating the JAK2/STAT3 pathway in porcine induced pluripotent stem cells (Wu et al., 2019). Moreover, JAK2/STAT3 signaling pathway has been found to be implicated in the pathogenesis of AS (Wang et al., 2020c; Wang et al., 2020d). For example, the inhibition of JAK2/STAT3 signaling by JAK2 inhibitor Ruxolitinib inhibited AS progression, alleviated lipid and calcium burdens, and weakened proinflammatory responses in AS rabbits (Yang et al., 2020).

Previous studies have suggested that m6A writers including METTL3 usually exert their functions through m6A readers (Jiang, et al., 2021; Robinson et al., 2019). For instance, METTL3 facilitated the tumorigenesis and metastasis of colorectal cancer by inhibiting YPEL5 expression in a YTHDF2 (an m6A reader)-dependent manner (Zhou et al., 2021). METTL3 inhibited the senescence of human mesenchymal stem cells by increasing the stability of MIS12 mRNA via the m6A reader IGF2BP2 (Wu et al., 2020). Hence, m6A readers that could mediate the regulatory effect of METTL3 on JAK2 were searched based on the bioinformatics prediction data and previous research outcomes. Among the m6A readers, IGF2BP1 was found to be the potential intermediate mediator of METTL3 and JAK2. IGF2BP1, also known as IMP1, is a member of the IGF2 mRNA-binding protein (IGF2BP) family (Huang et al., 2018). IGF2BPs have been identified as crucial players in the regulation of multiple biological processes (*e.g.* embryogenesis, cell migration, proliferation, and invasion) under normal and stress conditions (Degrauwe et al., 2016; Huang et al., 2018). Moreover, two recent studies suggested that IGF2BP1 was closely associated with the pathogenesis of AS (Li et al., 2019; Nguyen et al., 2018). For instance, Li *et al.* showed that the exosomes derived from mesenchymal stem cells (MSCs) hindered mouse AS development partly by reducing IGF2BP1 expression (Li et al., 2019).

Our present study demonstrated that IGF2BP1 could directly bind to an m6A site in the CDS region of JAK2 mRNA, resulting in the increase of JAK2 expression and JAK2 mRNA stability in HUVECs. Moreover, METTL3 knockdown attenuated the interaction between IGF2BP1 and JAK2 mRNA in HUVECs. These data suggested that METTL3 regulated JAK2 expression by IGF2BP1 in HUVECs. In line with our data, previous studies also demonstrated that METTL3 could influence the development of diseases by regulating the expression of some genes under the cooperation with m6A reader IGF2BP1 (Chen et al., 2021b; Liu et al., 2020b). For instance, Wei *et al.* demonstrated that METTL3 cooperated with IGF2BP1 to increase the stability of transcription factor-activating enhancer-binding protein 2C (TFAP2C) mRNA, while the increase of TFAP2C expression level potentiated the resistance to cisplatin in seminoma (Wei et al., 2020). Also, He *et al.* demonstrated that METTL3 enhanced the stability of SEC62 mRNA via IGF2BP1 in gastric cancer (He et al., 2019).

It has been reported that METTL3 overexpression could markedly weaken LPS-induced inflammatory responses in macrophages by inactivating the NF-κB signaling pathway (Wang et al., 2019). Moreover, the enforced expression of METTL3 could notably potentiate M1 macrophage polarization by directly methylating STAT1 mRNA (Liu et al., 2019). Additionally, a recent study demonstrated that METTL3 mediated proinflammatory responses in HUVEC endothelial cells, and METTL3 knockdown hampered AS progression in a mouse model (Chien et al., 2021). Given the close association of inflammation and AS (Kasikara et al., 2018; Bäck et al., 2019), we speculated sh-METTL3 might regulate both inflammatory cells and endothelial cells to protect against AS.

## Conclusion

Our data demonstrated that METTL3 knockdown alleviated ox-LDL-induced endothelial cell dysfunction, prevented *in vivo* angiogenesis of developing embryos, and hindered AS progression in mouse models through inhibiting JAK2/STAT3 pathway via m6A reader IGF2BP1, elucidating the vital roles of METTL3/JAK2/STAT3 axis in AS process along with IGF2BP1-dependent regulatory mechanisms. An in-depth insight into the m6A-related gene regulatory networks might contribute to the better management of m6A-associated diseases including AS. Also, we demonstrated that METTL3 knockdown could inhibit angiogenesis *in vitro* and *in vivo*, providing a potential therapeutic method for cardiovascular diseases.

## Data Availability

The datasets presented in this study can be found in online repositories. The names of the repository/repositories and accession number(s) can be found in the article/Supplementary Material.

## Supplementary Material

SUPPLEMENTARY TABLE 1 Genes with the notable reduction (|log2FoldChange| > 1 and *p* < 0.05) in the m6A methylation levels of mRNAs in METLL3 loss group versus the control group.

SUPPLEMENTARY TABLE 2 Genes that were markedly downregulated (|log2FoldChange| > 1 and *p* < 0.05) in METLL3 loss group versus the control group.

SUPPLEMENTARY TABLE 3 Common genes in Supplementary Tables S1, S2.

SUPPLEMENTARY TABLE 4 StarBase-predicted mRNAs that had a possibility to bind with METTL3.

SUPPLEMENTARY TABLE 5 Common genes in Supplementary Tables S1, S2, and S4.

SUPPLEMENTARY FILE 1 The formula of H10540 feed.

SUPPLEMENTARY FIGURE 1 HUVECs were treated with ox-LDL (10 μg/ml) for 24 h. Next, protein levels of JAK2, STAT3 and p-STAT3 were measured by western blot assay.

## Abbreviations

AS, Atherosclerosis; HDL-C, high-density lipoprotein-cholesterol; IGF2BP, IGF2 mRNA-binding protein; LDL-C, low-density lipoprotein-cholesterol; MSCs, mesenchymal stem cells; m6A, N6-methyladenosine; ox-LDL, Oxidized low-density lipoprotein; TC, total cholesterol; TG, triglyceride; TFAP2C, transcription factor-activating enhancer-binding protein 2C.
