# Supplementary figures and images for: N6-Methyladenosine Methyltransferase METTL3 Promotes Angiogenesis and Atherosclerosis by Upregulating the JAK2/STAT3 Pathway via m6A Reader IGF2BP1
Source: Front Cell Dev Biol. 2021 Dec 7;9:731810. doi: 10.3389/fcell.2021.731810 (PMC8689138; doi:10.3389/fcell.2021.731810)

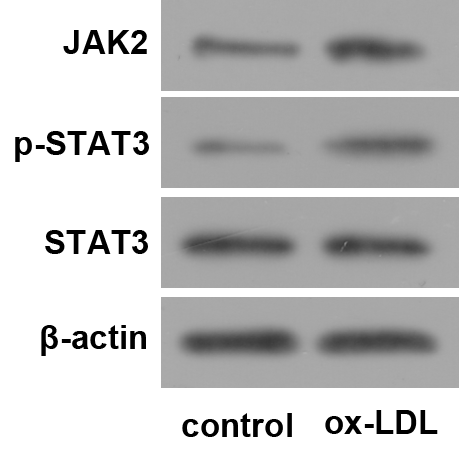

Supplement: Supplementary file 3 [file Image1.tif]
