# Supplementary material for: N6-Methyladenosine Methyltransferase METTL3 Promotes Angiogenesis and Atherosclerosis by Upregulating the JAK2/STAT3 Pathway via m6A Reader IGF2BP1
Source: Front Cell Dev Biol. 2021 Dec 7;9:731810. doi: 10.3389/fcell.2021.731810 (PMC8689138; doi:10.3389/fcell.2021.731810)
Supplement: Supplementary file 7 [file DataSheet1.docx]

**The formula of high-fat and high-cholesterol feed H10540**

| Component | g | kcal |
| --- | --- | --- |
| Casein | 222.88 | 891.52 |
| Cystine | 3.34 | 13.36 |
| Corn starc | 236.25 | 945 |
| Maltodextrin | 79.12 | 316.48 |
| Sucrose | 125.93 | 503.72 |
| Cellulose | 55.72 | 0 |
| Soybean oil | 27.86 | 250.74 |
| Cocoa butter | 172.73 | 1554.57 |
| Mineral mixture M1002 | 11.14 | 0 |
| Calcium hydrogen phosphate | 14.49 | 0 |
| Calcium carbonate | 6.13 | 0 |
| Potassium citrate | 18.39 | 0 |
| Vitamin mixture V1001 | 11.14 | 46.24 |
| Choline bitartrate | 2.23 | 0 |
| Cholesterol | 12.54 | 0 |
| Edible blue dye | 0.056 | 0 |
| Edible yellow dye | 0.056 | 0 |
| total | 1000 | 4521.63 |
